# Porous Polylactide Microparticles as Effective Fillers for Hydrogels

**DOI:** 10.3390/biomimetics8080565

**Published:** 2023-11-23

**Authors:** Yuriy D. Zagoskin, Yana E. Sergeeva, Yuliya S. Fomina, Daniil V. Sukhinov, Sergey N. Malakhov, Egor O. Osidak, Elena A. Khramtsova, Pavel M. Gotovtsev, Sergei N. Chvalun, Timofei E. Grigoriev

**Affiliations:** 1 National Research Centre “Kurchatov Institute”, 123182 Moscow, Russia; zagos@inbox.ru (Y.D.Z.); yanaes2005@gmail.com (Y.E.S.); ledy_uylia-98@mail.ru (Y.S.F.); suhinov.dv@phystech.edu (D.V.S.); malakhov_sn@nrcki.ru (S.N.M.); s-chvalun@yandex.ru (S.N.C.); timgrigo@gmail.com (T.E.G.); 2 Moscow Institute of Physics and Technology, National Research University, 141700 Dolgoprudny, Russia; 3 Imtek Ltd., 121552 Moscow, Russia; eosidak@gmail.com; 4 Dmitry Rogachev National Medical Research Center of Paediatric Haematology, Oncology and Immunology, 117198 Moscow, Russia; 5 N.M. Emanuel Institute of Biochemical Physics, Russian Academy of Sciences, 119334 Moscow, Russia; alyonushk@gmail.com

**Keywords:** collagen, hydrogel, polylactide, porous materials, scanning acoustic microscopy, C-phycocyanin

## Abstract

High-strength composite hydrogels based on collagen or chitosan–genipin were obtained via mixing using highly porous polylactide (PLA) microparticles with diameters of 50–75 µm and porosity values of over 98%. The elastic modulus of hydrogels depended on the filler concentration. The modulus increased from 80 kPa to 400–600 kPa at a concentration of porous particles of 12–15 wt.% and up to 1.8 MPa at a filling of 20–25 wt.% for collagen hydrogels. The elastic modulus of the chitosan–genipin hydrogel increases from 75 kPa to 900 kPa at a fraction of particles of 20 wt.%. These elastic modulus values cover a range of strength properties from connective tissue to cartilage tissue. It is important to note that the increase in strength in this case is accompanied by a decrease in the density of the material, that is, an increase in porosity. PLA particles were loaded with C-phycocyanin and showed an advanced release profile up to 48 h. Thus, composite hydrogels mimic the structure, biomechanics and release of biomolecules in the tissues of a living organism.

## 1. Introduction

Biomimetic hydrogels are promising as an extra-cellular matrix (ECM) in various fields of regenerative medicine: cartilage tissue engineering [1,2], wound healing [3], etc. Hydrogels may contain signal molecules and interact with cells, therefore mimicking the structure of the ECM with the required level of mechanical properties of native tissue [4]. Hydrogel materials can be based on natural (collagen, gelatin, fibrin, chitosan, hyaluronic acid, sodium alginate) and synthetic polymers (polyethylene glycol, polyvinyl alcohol). Despite the variety of natural materials used, almost every article and review reports that the mechanical properties of hydrogels are low compared to the ones of native tissues [5,6]. Two approaches to solving the problem have been proposed:

1. The development of synthetic hydrogels with dynamic covalent crosslinks [7] or brush structures [8] that perfectly mimic tissue mechanics. However, such materials are often non-resorbable, and thorough study of cells’ interactions with the scaffold is required.
2. The development of composite hydrogels based on a natural polymer filled by a biocompatible filler containing active biomolecules. For example, a novel injectable nanocomposite was designed as a sustained-release platform for cartilage regeneration through the integration of a chitosan-based hydrogel, articular cartilage stem cells (ACSCs) and mesoporous SiO_2_ nanoparticles loaded with anhydroicaritin (AHI) [9].

In this study, we propose an approach to create composite hydrogels based on thermosetting collagen hydrogels and chemically cross-linked chitosan–genipin hydrogels. Two of the most used in tissues engineering polymers—collagen and chitosan—were chosen. To adjust their mechanical properties, they were paired with fillers—porous polylactide particles were utilized. To mimic the biochemical release of natural materials, the bioactive compound of C-phycocyanin was added. Now let’s look at the base polymers and hydrogels in more detail.

### 1.1. Collagen and Collagen Gels

Type I collagen is the primary component of the extracellular matrix for most living tissues. Collagen biomatrices are widely used in clinical practice due to their biocompatibility, low toxicity and weak immune reactions [10]. Collagen molecules in the implanted carrier eventually degrade into simpler compounds via the action of specialized enzymes, which are excreted from the body, taking an active part in biosynthesis at the cellular level and stimulating reparative processes, including the formation of the body’s own collagen over time.

Collagen in the form of a hydrogel is widely presented in the human tissues. Therefore, collagen hydrogels themselves serve as a bioresorbable substrates on which host cells can migrate after implantation, restoring the functionality of damaged tissue [11]. Usually, they are formed from cooled acid-solubilized type I tropocollagen upon neutralization and heating. But, due to their low strength properties, such materials have limited applications. Collagen hydrogels, on the other hand, can act as carriers for the controlled delivery [12] and localization of growth factors [6] and other biologically active substances.

### 1.2. Chitosan and Chitosan Gels

Chitosan is a natural polysaccharide containing reactive amino groups [13]. Chitosan hydrogels are already used as biomimetic structures [14], including those with hydroxyapatite filler for cartilage regeneration [15]. Amino groups allow chitosan to enter into a crosslinking reaction with natural biocompatible genipin [16]. Chitosan–genipin gels attract the attention of researchers due to their excellent biocompatibility [17] and can be the basis for composite structures [18].

### 1.3. Composite Gels with Porous Microparticles

A number of approaches are used to modify the mechanical properties of hydrogels: adjusting the cross-linking density of polymer chains, macromolecule concentration, and forming composite hydrogel materials by filling them with various more rigid components. Earlier obtained porous polylactide microparticles were chosen as a filler. Microparticles increase the mechanical properties of chitosan and collagen hydrogels and are promising as potential osteoplastic materials [19]. They prolong the release of growth factors such as bone morphogenetic protein (BMP-2) for up to 6 days and have osteoinductive potential with low doses of BMP-2. In addition, it was shown that creating gene-activated matrices via the use of such porous particles is possible. They can serve as effective containers for polyplexes and adenovirus-based gene BMP-2 [20].

### 1.4. C-Phycocyanin

The impregnation of bioactive compounds is one of the most effective routes of biofunctionalization. Phycocyanin is one of the promising bioactive agents. C-Phycocyanin derived from cyanobacteria (C-PC) is of interest as a potential excipient. C-PC is a bright blue water-soluble, fluorescent pigment–protein complex of light-harvesting antenna complexes of photosynthetic organisms (cyanobacteria, rhodophytes and cryptomonads). The main commercial source of C-PC is the filamentous alkophilic cyanobacteria *Arthrospira platensis*. According to forecasts [21], its global marketing value is expected to reach USD 279.6 million in 2030. Such high interest is due to the various biological functions and proven therapeutic effects of this compound. C-PC has antioxidant, anti-inflammatory, antitumor, antiviral, immunostimulating, wound healing, antidiabetic and other therapeutic properties [22,23,24,25]. C-PC has many biotechnological applications in the food, cosmetic, pharmaceutical and medical industries.

It should be noted that C-PC is sensitive to a number of factors, such as pH, light, temperature, etc. [26]. At the moment, to prevent or reduce the rate of C-PC degradation, as well as to increase its stability and bioavailability, different methods have been proposed for encapsulating C-PC in various shells [27,28], which, upon dissolution or wetting, will initiate the process of C-PC release. At the same time, it is possible to change the kinetics of C-PC release by varying the parameters of the shell (composition and thickness, porosity, solubility of the material).

Different methods for obtaining C-PC-containing liposomes [29], santosomes [30], hyalurosomes [31,32], chitosomes [33], microcapsules [34], nanoparticles [35,36], hydrogels [37,38,39], gel beads [40,41] and structured phycocyanin-loaded micro/nanofibrous [42] have been described in the literature.

Thus, C-PC is considered to be a promising agent for the treatment of inflammatory and oxidative diseases, wound healing and tissue regeneration. And its inclusion in the polymer matrix allows us to regulate the release rate, prolonging its effect via oral drug administration and the external use of medicines containing C-PC.

In this study, we focused on the development of a new material, consisting of a biomimetic polymeric hydrogel filled with porous polylactide microparticles, which provide the long-term release of the therapeutic agent C-PC.

## 2. Materials and Methods

### 2.1. Materials

#### 2.1.1. Porous Polylactide Microparticles

Porous microparticle preparation from 2 wt.% of polylactide (4032D, Corbion, Lenexa, KS, USA) in 1,4-dioxane was conducted. The solution was sprayed into the liquid nitrogen, and then samples were lyophilized using a Martin Christ ALPHA 2-4LSC freeze-dryer.

#### 2.1.2. Highly Concentrated Collagen Gel

Lyophilized sterile atelocollagen type I from porcine tendons (Viscoll; Imtek Ltd., Moscow, Russia) was reconstituted in 10 mM acetic acid to a concentration 100 mg/mL.

Preparation of gel: collagen gel was prepared via the incubation of acidic collagen solution in ammonia vapors at 37 °C for 30 min.

#### 2.1.3. Chitosan–Genipin Hydrogels

Dry genipin (GL8880, Glentham Life Sciences, Corsham, UK) was added to a solution of chitosan (ChitoClear 43000—HQG 10, Primex, Siglufjordur, Iceland) at a concentration of 2 wt.% in 0.1 M acetic acid to obtain hydrogels. The concentration of the crosslinker was 7 wt.% calculated to the dry chitosan. The materials were kept in a closed container at room temperature for at least 48 h until the gelation process was completed after mixing the components.

#### 2.1.4. Composite Hydrogels

Polylactide particles were added to chitosan or collagen solutions until crosslinking and mixed with a narrow spatula created a homogeneous system.

#### 2.1.5. C-Phycocyanin

C-phycocyanin (C-PC) was obtained via exhaustive extraction from the cyanobacteria *Arthrospira platensis* biomass. The strain *Arthrospira platensis* B-12619 was provided by the Russian National Collection of Industrial Microorganisms. The cyanobacteria was cultivated in modified Zarrouk medium [43]. The cultivation was performed in 500 mL Erlenmeyer flasks containing 200 mL of the culture medium in a Innova 42R shaker–incubator (New Brunswick, Eppendorf, Hamburg, Germany) (30 °C, 140 rpm, continuous lighting and PAR at 16 ± 2 μmol/(m^2^·s)). The cyanobacteria biomass was harvested at the end of the exponential growth phase via centrifugation (12300× *g*, 15 min, 25 °C), washed twice with distilled water and stored at −20 °C.

Crude C-PC was extracted from pretreated (threefold freezing/thawing cycle) wet biomass, as described in [44]. The C-PC crude extracts were purified via ammonium sulfate precipitation. Ammonium sulfate was gradually added to crude extracts to achieve 20, 40 and 60% saturation with continuous stirring. In the last stage (60% ammonium sulfate saturation), the C-PC precipitate was dissolved in deionized water and dialyzed. Then, the dialyzed C-PC was freeze-dried and stored in the dark at 4 °C.

The C-PC concentration was calculated according to [45], and the C-PC purity index (PI) was determined according to [46].

C-PC (with PI over 3.5) was loaded into the PLA microparticles by adding 1.5 mL of an aqueous C-PC solution (1.06 ± 0.02 and 2.04 ± 0.05 mg/mL) to the microparticles (50.95 ± 0.21 mg). The mixture was vacuumed several times until homogeneity was reached. Subsequent freeze-drying led to further C-PC adsorption on the surface and in the pores of PLA granules. The PLA/C-PC microparticles were stored at 4 °C in the dark until further testing.

To study the in vitro release of loaded C-PC from the PLA/C-PC microparticles, PLA/C-PC microparticles (15.60 ± 0.14 mg) were suspended in 2 mL saline (pH 7.4) and placed in a shaker–incubator (37 °C, 100 rpm) for 24 h in the dark. During the release process, aliquots of the supernatant (400 μL) were periodically collected and replaced by the same volume of fresh saline.

The C-PC release amount was obtained by considering the cumulative quantity of C-PC based on the absorption spectra and determination of C-PC concentration. All measurements were performed in triplicate.

### 2.2. Experimental

#### 2.2.1. Scanning Electron Microscope

Material morphology was studied using a Phenom XL desktop scanning electron microscope (Thermo Fisher Scientific, Waltham, MA, USA) with a backscattered electron detector at an accelerating voltage of 5 kV and a residual pressure of 10 Pa without applying any conductive coating to the samples.

#### 2.2.2. Optical Microscopy

Received materials were studied using an Axio Imager.M2m optical microscope (Carl Zeiss, Baden-Württemberg, Germany). A thin slice of the sample was put between two glass plates and viewed in crossed polarized mode at 5× magnitude.

#### 2.2.3. Ultrasound Investigation

Scanning acoustic microscopy is a promising non-invasive instrument for investigations of non-transparent biomedical samples and soft tissues [47]. The experiment was carried out using impulse acoustic microscope SIAM-2011 (developed in the Laboratory of Acoustic Microscopy at the Institute of Biochemical Physics, Russian Academy of Science) with a 15 μm scanning step, equipped with an acoustic lens of 200 MHz and an angle aperture of 11°. The bidistilled water was used as an immersion liquid. Acoustic images were analyzed using ImageJ software (version 1.54f).

#### 2.2.4. Mechanical Properties

The study of mechanical characteristics of sponges was carried out via the universal test machine Instron-5965 (Instron, Norwood, MA, USA) under uniaxial compression. The tests were carried out at room temperature and 60% relative humidity. The sample size was 5 × 5 × 10 mm. The deformation rate was 10 mm/min. The modulus of elasticity was determined via the tangent of the angle of inclination of the secant in the range of 0–10% deformation. Statistical sampling of three samples of each type was performed.

#### 2.2.5. Fourier Transform Infrared Spectroscopy (FTIR)

IR spectra were recorded using a Nicolet iS5 IR Fourier spectrometer (Thermo Fisher Scientific, Waltham, MA, USA) with an iD5 ATR accessory (crystal–diamond). The spectral range was 4000–550 cm^−1^, the spectral resolution was 4 cm^−1^ and the number of scans was 32. The spectra were recorded and processed using the standard software of the instrument (Omnic 8.2).

### 2.3. Statistical Analysis

All data were processed using the software package OriginLab PRO (Origin 2023 (10.0)). Experimental result were presented with standard deviation.

## 3. Results and Discussion

### 3.1. Structure Polylactide Particles

**Figure 1:**
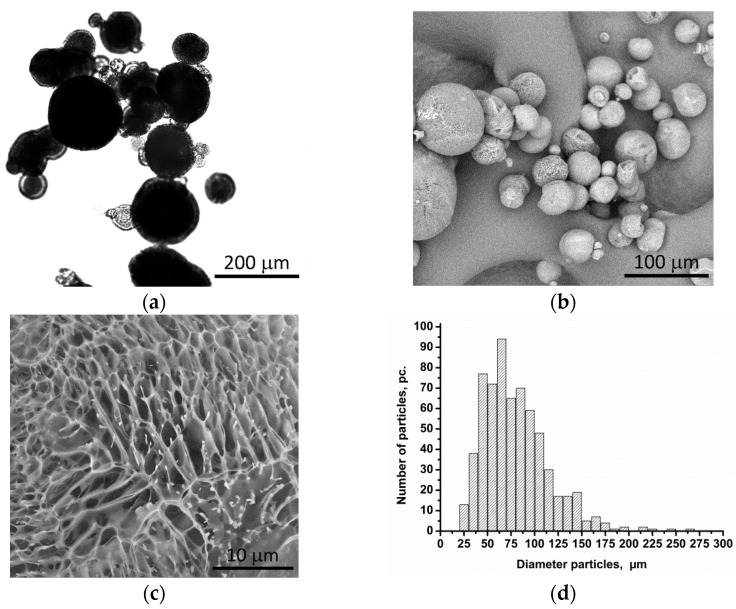
Optical and electron micrographs of (**a**,**b**) particles and (**c**) surfaces and (**d**) particle size distribution.

The resulting porous polylactide particles have a spherical morphology with an average diameter of 50–75 µm (Figure 1). The particle structure is porous, with interpenetrating pores with an average diameter of up to 10 µm. The total porosity of materials is over 98%.

### 3.2. C-PC-Loaded Polylactide Particles

As mentioned earlier, C-PC has high antioxidant activity. It has been proven that, due to its unique properties, C-PC has a positive effect on the treatment of diseases of various etiologies, including in the processes of skin healing and tissue regeneration. That is why C-PC was chosen as a model of bioactive molecules for our investigation. The therapeutic grade C-PC, with initial concentrations of 1 and 2 mg/mL, was incorporated into the polylactide particles (hereinafter, PLA/CPC-1, PLA/CPC-2). The choice of tested concentrations was determined using the literature’s data on the therapeutic concentrations of C-PC [37,39,48], as well as our own results [44].

**Figure 2:**
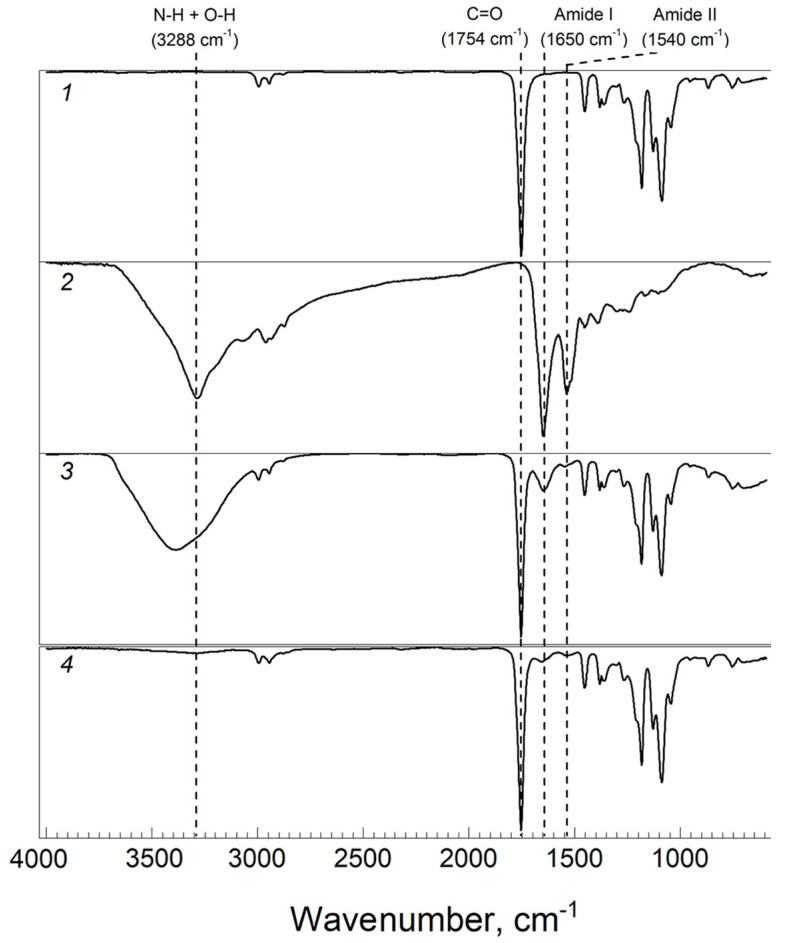
FTIR-ATR spectra: 1—PLA particles; 2—C-PC powder; 3—PLA particles with C-PC; 4—PLA particles after C-PC release.

For both the initial PLA particles and lyophilized powder C-PC, PLA particles loaded with C-PC were investigated via Fourier-Transform infrared spectroscopy (FTIR) (Figure 2).

The IR spectrum of PLA particles shows that the strong band at 1754 cm^−1^ corresponds to C=O bond stretching, bands at 2995 cm^−1^ and 2945 cm^−1^ are assigned to C–H stretching of –CH_3_ and bands at 1450–1350 cm^−1^ are assigned to C–H bending vibrations. The most characteristic absorption of ester C–O stretching is at 1088 cm^−1^. The FTIR spectrum of PLA corresponds to the spectra reported in literature [42]. It should be noted that in the IR spectrum of PLA, the band at 956 cm^−1^ refers to the absorption of the amorphous phase, and at 920 cm^−1^, it refers to the crystalline one [49]. Since the band at 920 cm^−1^ in the IR spectrum of PLA particles has near-zero intensity, we can conclude that the polymer in the particles is in an amorphous state (while the original granular PLA is characterized by a degree of crystallinity of about 40%).

The IR spectrum of C-PC contains specific bands of amide I (C=O stretching vibrations) and amide II (N–H bending and C–N stretching) at 1650 cm^−1^ and 1536 cm^−1^, respectively, and the intensity of the Amide I band is higher than Amide II. The position and shape of the amide I band is used to analyze the secondary structure of the protein. The sharp peak of the amide I band points to the α-helix as the main element of its secondary structure [50]. C-PC alone shows a broad peak from 3000–3500 cm^−1^, which represent the O–H stretch of acid. It should be noted that the IR spectrum of lyophilized pure C-PC also confirmed the absence of impurities of inorganic sulfates and phosphates (absence of intense bands in the region of 1040 and 1015 cm^−1^). The inclusion of C-PC in PLA particles was confirmed by the presence of sharp peak of amide I band at 1651 cm^−1^ and amide II at 1549 cm^−1^ in PLA/C-PC particles. It is interesting that, compared to C-PC, PLA/C-PC particles had a wider band at 3000–3500 cm^−1^.

### 3.3. C-PC Release from the PLA Particles

The release of C-PC from various polymer matrices into solutions over a wide pH range has been described in the literature. It should be noted that C-PC is a protein-chromophoric complex that is very sensitive to heat and pH fluctuations. At an acidic pH, a change in the native conformation of the protein molecule occurs, which leads to a change in the spatial structure of the chromophore (phycocyanobillin) and a disruption of the native properties of this compound, including a decrease in antioxidant activity.

**Figure 3:**
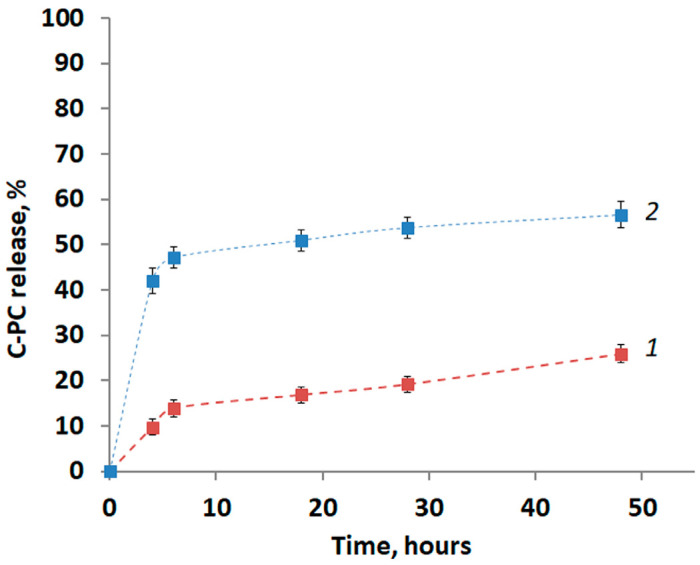
In vitro release profiles of C-PC from PLA particles: 1—PLA/CPC-1; 2 PLA/CPC-2.

In the present study, in vitro C-PC releases in an environment with physiological pH (pH 7.4) were determined. The release curves of C-PC from PLA particles are shown in Figure 3.

The continuous release process of C-PC can be divided into several phases. During the first 4 h, initial burst release (especially at the higher concentration) was observed (Figure 3). The rapid C-PC release could attributed to C-PC loaded at or near the surfaces of PLA particles. At the second stage (4–6 h), the release process was slowed down, and the next phase (from 6 h) was characterized by the stable release of C-PC, which may be due to the release of C-PC from the pores.

Regardless of the concentration of the initial solution used to load the particles, the main amount of C-PC is released in the first 6 h: over 50% of the total was released for PLA/CPC-1 and more than 80% for PLA/CPC-2. Release can occur up to 48 h, as can be seen from the curves.

Thus, more than 50% of the initial amount of C-PC was released from PLA/CPC-2. And about 25% was released after 48 h from PLA/CPC-1.

The FTIR spectrum after the C-PC release is very similar to the spectrum of native PLA particles (Figure 2). This study revealed that C-PC is non-covalently attached to PLA particles.

Thus, PLA particles could be used as the polymer matrix for C-PC with sustaining release in physiological pH environment.

### 3.4. Structure of Composite Hydrogels

#### 3.4.1. Collagen–PLA Microparticle Composite Hydrogels

**Figure 4:**
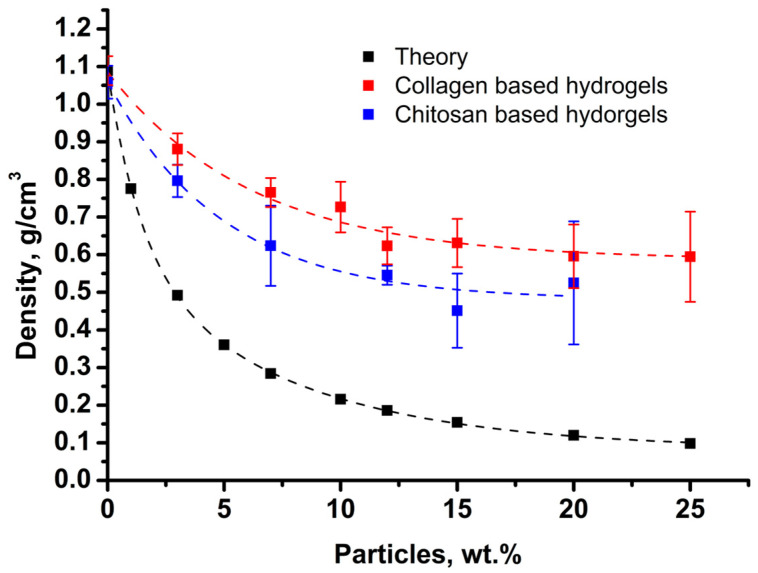
Density of the composite material with various contents of porous polylactide particles in hydrogels.

Hydrogels based on a 5 wt.% solution of collagen in a slightly acidic medium or chitosan 2 wt.% solution in 0.1 M acetic acid were filled with porous polylactide microparticles. The mass contents of particles ranged from 1 to 25 wt.%. Since particles are deformed during sample preparation, it is impossible to reliably establish the volume fraction of the particles. However, the total density of the composite material was determined, which decreases with the increasing number of particles (Figure 4). It can be seen that with the increase in the particle content, the density of the material decreases at a noticeably slower rate compared to the model of absolutely incompressible spheres. Nevertheless, the decrease in density below 1.0 g/cm^3^ indicates the incomplete wetting of the porous particles and their air contents.

Three-dimensional data (C-scans) were obtained via the ultrasonic analysis of particle distribution in the volume of samples. For each sample from the C-scans, three regions of interest (ROI) in the focal zone were selected, avoiding the edge regions of the sample. ROI was 2 × 2 mm, and a thickness of 77 μm was measured.

**Figure 5:**
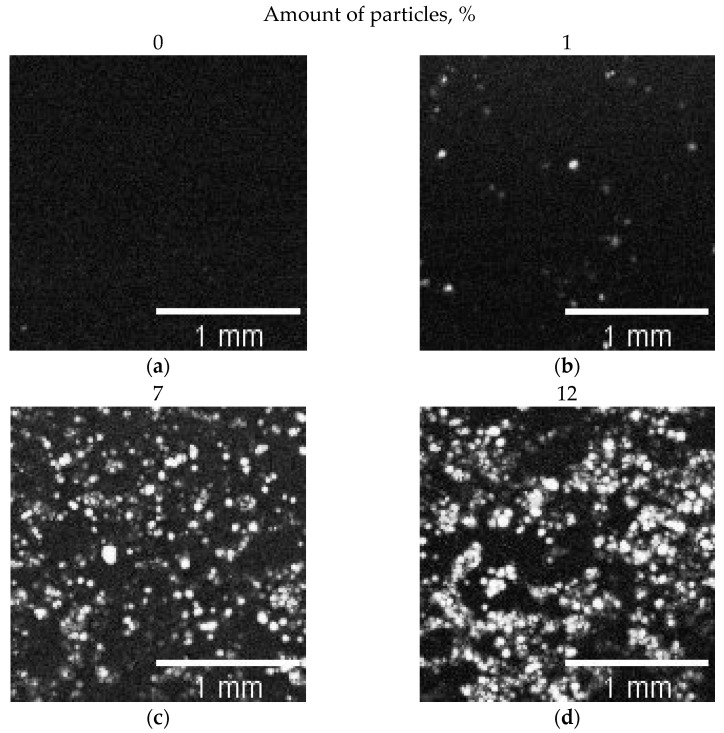
Acoustic images of the internal microstructure of the (**a**) pure collagen sample (amount of particles 0%) and the (**b**–**d**) composite hydrogels with different numbers of particles.

Due to the high echogenicity of the air filling the spongy polylactide particles, the high acoustic contrast of the particles in the ultrasound images is provided (Figure 5). Using the ImageJ software, ROI were analyzed to determine the area of the image occupied by particles with air (Table 1). The empty collagen gels were used as a control.

**Figure 6:**
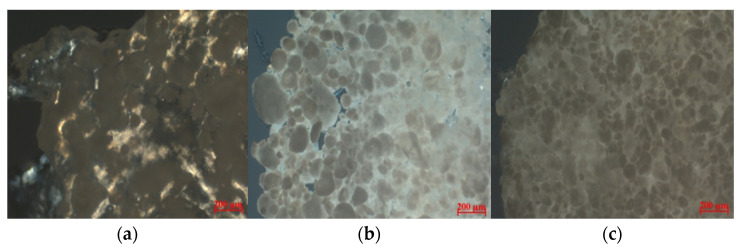
Optical micrographs of collagen hydrogels filled with porous microparticles; particle concentrations: (**a**) 1.0 wt.%; (**b**) 12 wt.%; (**c**) 25 wt.%.

The optical photograph shows (Figure 6) that with increasing particle concentration, the particles become denser. Moreover, even at 1 wt.% of particles, their engagement is observed, which determines the increase in mechanical characteristics. The difference between crossover concentration obtained via optical and acoustic microscopy is probably caused by different slice thicknesses.

#### 3.4.2. Chitosan–PLA Microparticle Composite Hydrogels

**Figure 7:**
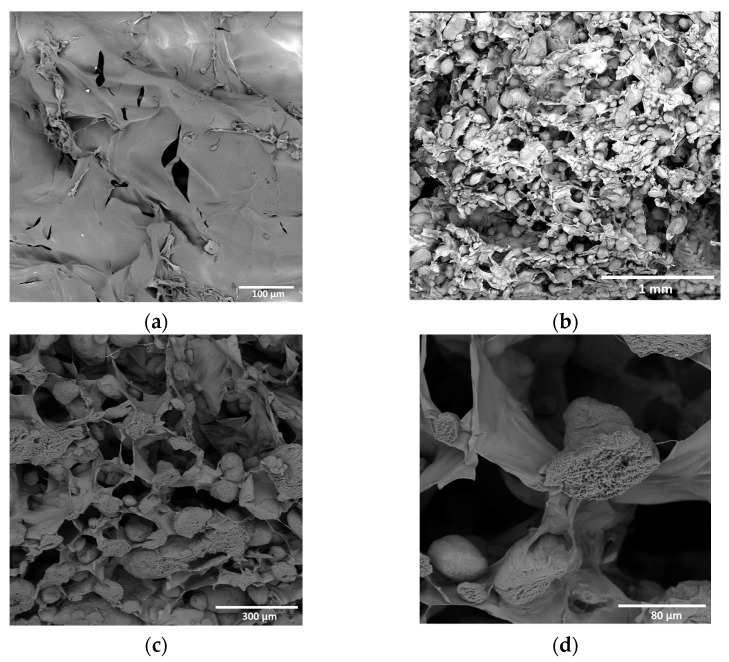
SEM images: (**a**) chitosan–genipin hydrogel; (**b**–**d**) chitosan–genipin hydrogel with porous microparticles.

SEM micrographs of freeze-dried composite hydrogels based on chitosan–genipin with PLA particles demonstrate a good distribution of particles in the matrix (Figure 7). In 1000× magnification images, one can see clearly the internal porous structure of the sections of the particles.

### 3.5. Mechanical Properties of Collagen and Chitosan Hydrogels Filled with Porous Lactide Nanoparticles

**Figure 8:**
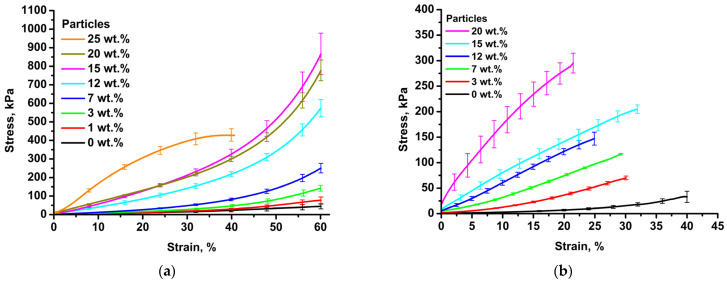
Averaged compression curves for collagen-based (**a**) and chitosan-based (**b**) composite hydrogels and porous polylactide particles.

According to the results of mechanical tests for all the materials studied, curves in stress–strain coordinates were obtained. The averaged dependencies are shown in Figure 8.

**Figure 9:**
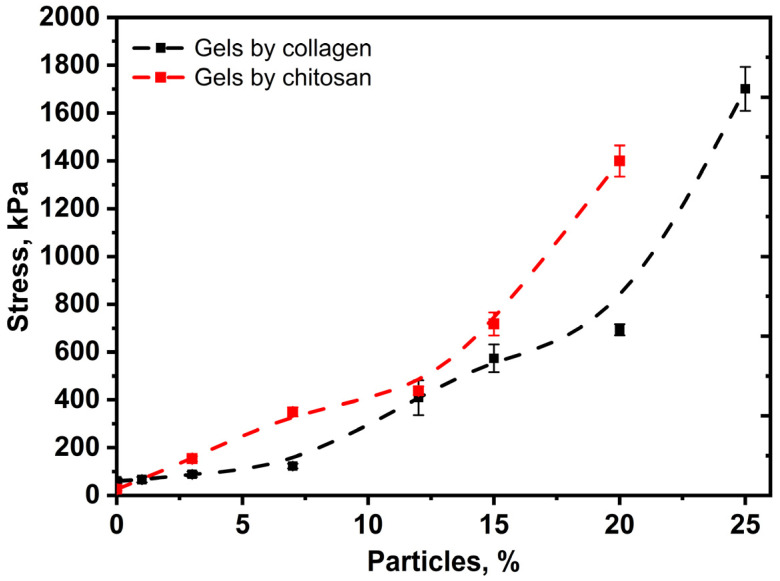
Dependence of elasticity moduli on particles in chitosan and collagen hydrogels.

For all the samples studied, the stress changes nonlinearly with strain. For up to 20 wt.% of particle content, the curves look qualitatively similar; for up to 20% of compression, there is a long linear stretch of elastic deformation, which turns into a smooth nonlinear rise associated with the compaction of the material. The elastic moduli were calculated from the slope to the initial part of the curves. As the particle content increases, the elastic modulus of materials increases according to the sigmoidal dependence (Figure 9). It can be seen that when deformation exceeds 25%, the compression curve of the sample with a particle content of 20% dips “below” the curve of the sample with a content of 15%. This may be due to the micro-delamination of the gel from the surface of the particles (although there is no visible destruction of the sample). The growth in the elastic modulus with particle concentration is associated with the formation of a continuous phase of overlapping particles and an increase in the density of the material. In this case, high particle porosity is important, since network formation occurs at a much lower particle content in the hydrogel phase compared to non-porous fillers. The elastic moduli lie in a wide range of values from 75 kPa to 900 kPa, which covers a wide range of values of native tissues. For example, the elastic modulus of breast tissue is 2–66 kPa, liver tissue is 10–390 kPa, gallbladder tissue is 250–641 kPa, urinary bladder tissue is 250 kPa, and uterus tissue is 20–1400 kPa [51].

There is a particular need to describe the sample with 25% particle content, for which the stress–strain dependence is extreme, with a maximum of 38–39% compression. Apparently, with this content, the continuity of the hydrogel phase is broken, and the deformation mechanism changes from elastic to quasi-plastic. Such a change in mechanical behavior is accompanied by a sharp increase in the modulus of elasticity and the appearance of deformation of the material fracture in the studied range. We also observe an increase in the elastic modulus and a smooth transition from elastic to quasi-plastic behavior in the case of chitosan–genipin hydrogels.

## 4. Conclusions

The filling of collagen and chitosan–genipin hydrogels with porous polylactide particles leads to an increase in the elastic modulus of the system while maintaining elasticity, which allows them to be used in a wide range of loads. Polylactide-based porous particles act as universal filler for various gelling systems. The filling effect is most pronounced for hydrogels, since polylactide is a hydrophobic polymer, and this allows non-wettable granules based on it to impart porosity to the gel–particle system.

Using acoustic microscopy in this study made it possible to visualize the homogeneity of composite gel volume and non-invasively determine the concentration at which contact interactions occur between the polylactide particles.

On the other hand, it was shown that porous polylactide particles can be an effective carrier for C-PC with a prolonged release profile. The combination of two approaches—the use of porous particles both as a modifier of strength characteristics and a carrier for a biologically active substance—made it possible to develop a biomimetic composite hydrogel that mimics the structure, biomechanics and biomolecular release in the tissues of a living organism.

## Figures and Tables

**Table 1:** Average of particles in % for composite collagen hydrogels with polylactide spongy particles.

% of Particles in Composite	% of Image Filled with Particles
0	0
1	1 ± 0.3
7	22 ± 0.2
12	36 ± 1

## Data Availability

Data is contained within the article.
